# The impact of environmental factors and contaminants on thyroid function and disease from fetal to adult life: current evidence and future directions

**DOI:** 10.3389/fendo.2024.1429884

**Published:** 2024-06-19

**Authors:** Maria E. Street, Anna-Mariia Shulhai, Maddalena Petraroli, Viviana Patianna, Valentina Donini, Antonella Giudice, Margherita Gnocchi, Marco Masetti, Anna G. Montani, Roberta Rotondo, Sergio Bernasconi, Lorenzo Iughetti, Susanna M. Esposito, Barbara Predieri

**Affiliations:** ^1^ Department of Medicine and Surgery, University of Parma and Unit of Pediatrics, University Hospital of Parma, Parma, Italy; ^2^ Unit of Paediatrics, University Hospital of Parma, P. Barilla Children’s Hospital, Parma, Italy; ^3^ Microbiome Research Hub, University of Parma, Parma, Italy; ^4^ Unit of Pediatrics, University Hospital of Modena, Department of Medical and Surgical Sciences for Mothers, Children and Adults, University of Modena and Reggio Emilia, Reggio Emilia, Italy

**Keywords:** thyroid, thyroid hormones, environmental pollution, food pollution, endocrine disruptors, COVID-19

## Abstract

The thyroid gland regulates most of the physiological processes. Environmental factors, including climate change, pollution, nutritional changes, and exposure to chemicals, have been recognized to impact thyroid function and health. Thyroid disorders and cancer have increased in the last decade, the latter increasing by 1.1% annually, suggesting that environmental contaminants must play a role. This narrative review explores current knowledge on the relationships among environmental factors and thyroid gland anatomy and function, reporting recent data, mechanisms, and gaps through which environmental factors act. Global warming changes thyroid function, and living in both iodine-poor areas and volcanic regions can represent a threat to thyroid function and can favor cancers because of low iodine intake and exposure to heavy metals and radon. Areas with high nitrate and nitrite concentrations in water and soil also negatively affect thyroid function. Air pollution, particularly particulate matter in outdoor air, can worsen thyroid function and can be carcinogenic. Environmental exposure to endocrine-disrupting chemicals can alter thyroid function in many ways, as some chemicals can mimic and/or disrupt thyroid hormone synthesis, release, and action on target tissues, such as bisphenols, phthalates, perchlorate, and per- and poly-fluoroalkyl substances. When discussing diet and nutrition, there is recent evidence of microbiome-associated changes, and an elevated consumption of animal fat would be associated with an increased production of thyroid autoantibodies. There is some evidence of negative effects of microplastics. Finally, infectious diseases can significantly affect thyroid function; recently, lessons have been learned from the SARS-CoV-2 pandemic. Understanding how environmental factors and contaminants influence thyroid function is crucial for developing preventive strategies and policies to guarantee appropriate development and healthy metabolism in the new generations and for preventing thyroid disease and cancer in adults and the elderly. However, there are many gaps in understanding that warrant further research.

## 1 Introduction

The main function of the thyroid gland is to produce triiodothyronine (T3) and thyroxine (T4). Thyroid hormones are key regulators of basal energy expenditure and metabolism, regular development and differentiation of cells, neurodevelopmental processes in the first years of life, linear growth, body composition and weight.

Normal thyroid function is regulated by the hypothalamic thyrotropin-releasing hormone (TRH) that stimulates the release of the thyroid-stimulating hormone (TSH) from the pituitary gland, which in turn drives synthesis and secretion of the thyroid hormones from the thyroid gland (1). The development of the thyroid gland begins around the third week of gestation, originating from an evagination of the pharyngeal floor in correspondence with the second branchial arch. At the end of the 7^th^ week of gestation, the embryonic thyroid is in its final position in front of the trachea. The production and secretion of thyroid hormones into the blood starts as early as the 10^th^- 12^th^ week of gestation (2). Since prenatal life, normal thyroid hormone production plays a key role in several physiological functions, such as the growth and development of the organism, including neurological and cognitive functions (3). In newborns, changes and defects in thyroid anatomy and function are related with pathological conditions, the most common being congenital hypothyroidism (CH), affecting 1 in 3,000 newborns (4); thyroid dysgenesis (agenesis, hypoplasia, and ectopy), thyroid dyshormonogenesis, hypothalamic-pituitary axis alterations and thyroid hormone resistance are all observed in this condition. Neonatal hyperthyroidism instead is associated mainly with Graves’ disease in mothers because of the transplacental passage of Thyrotropin-Receptor antibodies (TRAb) (5).

In children, acquired hypothyroidism is caused primarily by autoimmune thyroiditis with a current prevalence of 1–2% among children and adolescents (6), whereas the most frequent cause of hyperthyroidism is represented by Graves’ disease, more common in girls than in boys, with an incidence of 0.1 in 100,000 children and 3 in 100,000 adolescents (7). Low iodine availability can cause acquired hypothyroidism (8).

Thyroid cancers are less common in children than in adults, but it has been estimated that 1.8% of thyroid malignancies diagnosed in the US are in people aged less than 20 years. In recent years, a significant increase in pediatric thyroid cancers has been reported, in particular papillary thyroid carcinoma (PTC) and follicular thyroid carcinoma (FTC), whereas anaplastic (ATC), medullary (MTC), and poorly differentiated (PDTC) thyroid carcinomas are rarer (9).

In adults, thyroid diseases represent the most frequent endocrine disorders. The global prevalence of autoimmune diseases in pediatric age is about 5%. Among these, the most frequent autoimmune diseases are represented by autoimmune thyroid diseases, which include both Graves’ disease (GD) and Hashimoto’s thyroiditis (HT) (10). The epidemiological data of hypothyroidism ranges from 0.2% to 5.3% in Europe and from 0.3% to 3.7% in the USA, and is frequently associated with iodine deficiency and autoimmune thyroiditis. The prevalence of hyperthyroidism instead is similar in Europe (0.7%) and in the USA (0.5%). Graves’ disease is the major cause of hyperthyroidism in adults as in children, followed by toxic multinodular goiter and thyroid adenoma (11). Several studies have identified the importance genetic predisposition. Despite this, 20–25% of the phenotypic variation in autoimmune thyroiditis is due to environmental and/or epigenetic factors that also may be influenced by the environment (12).

All the different thyroid conditions mentioned so far have shown changes in their prevalence over time; this has suggested an important effect on the environment, including pollution and contaminants. Climate change, air and water pollution, nutrition and infectious agents can play an important role both in prenatal and postnatal life, influencing endocrine functions. Several studies have been conducted with the aim to investigate the role of environmental factors on thyroid function.

The synthesis, transportation and metabolism of thyroid hormones can be modified by toxic agents, that can interfere with hormone receptors and allow the beginning of autoimmune processes.

Also, air pollution exposure can cause thyroid diseases, and studies have considered the effects of carbon monoxide (CO), nitrogen dioxide (NO_2_), sulfur dioxide (SO_2_), ozone (O_3_), and particulate matter <2,5 μm (PM_2.5_), <10 μm (PM_10_) (13).

There is, in addition, a growing interest in endocrine-disrupting chemicals (EDCs) due to their wide use and pervasive presence in nature. All EDCs can change endocrine functions, as they can mimic or interfere with the normal functioning of the endocrine system and play a crucial role in regulating various physiological processes in the human body, including thyroid function (14, 15). EDCs include industrial agents (dioxins, polychlorinated biphenyls (PCBs) and alkylphenols), agricultural agents (herbicides, pesticides, fungicides, and insecticides), bisphenols (BPA, BPS, BPF), phthalates, drugs (ketoconazole, mitotane, cardiac glycosides, carbamazepine and nitrofurans) and heavy metals (cadmium, arsenic, nickel, beryllium and chromium) (16), and they are related to potential interference with normal thyroid function, and exposure is associated with a higher prevalence of thyroid diseases (17, 18).

The aim of this narrative review is to explore and consider comprehensively how environmental factors, such as the above-mentioned environmental pollution, climate change, EDCs, diet and micro-nutrients, and infectious agents influence thyroid function and disease. At variance with previous studies, we have focused also on the available evidence in relationship with the different ages of life, in particular, the prenatal, perinatal, infancy, adolescence, and adult periods of life were considered.

## 2 Materials and methods

### 2.1 Literature search strategy and selection criteria

**Figure 1:**
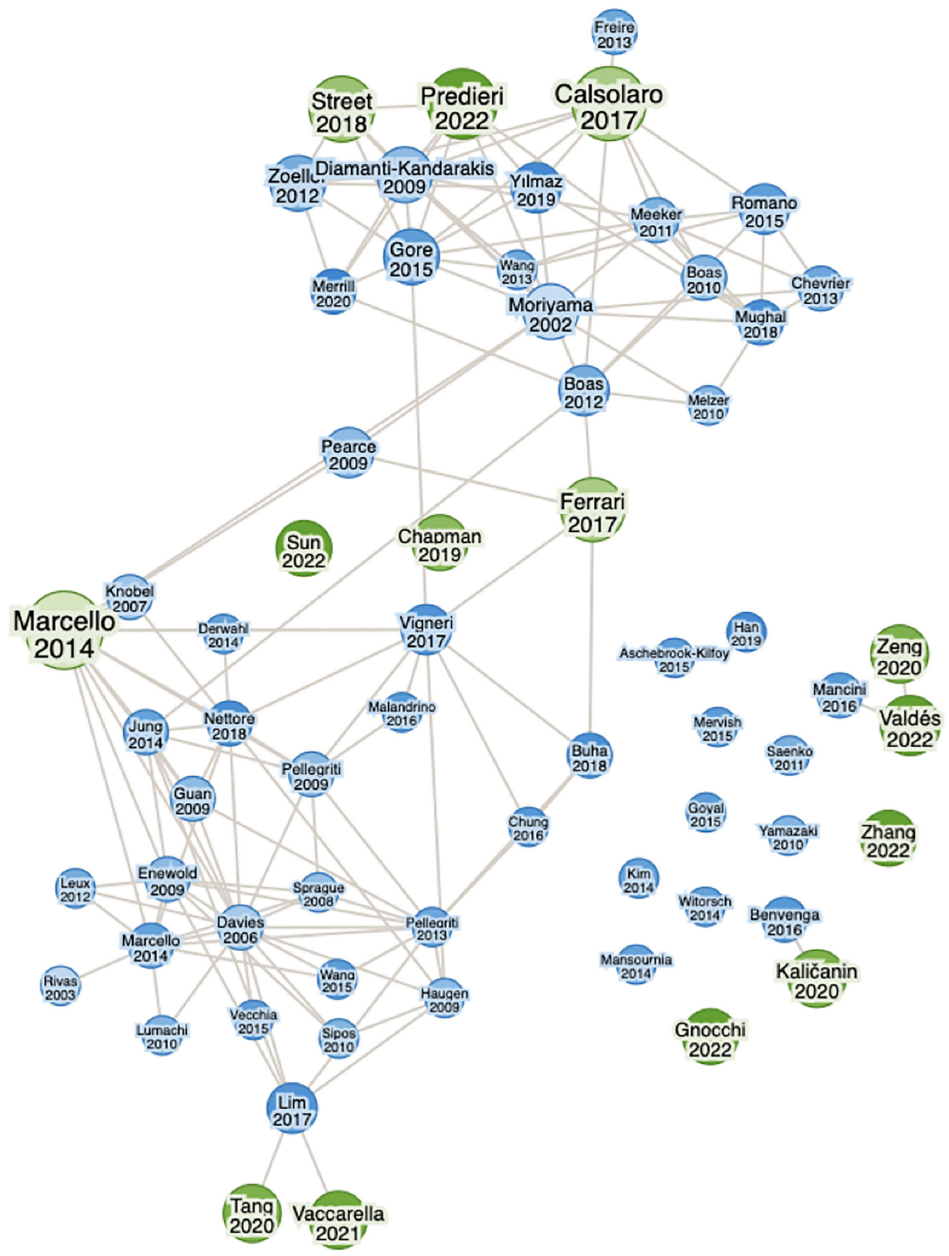
Literature map of key papers created with researchrabbitapp.com.

The research of the literature was done using specific research strings in Pubmed, Scopus, and Mendeley, via MeSH using specific keywords. The Cochrane Library database and all bibliographic sources available on scientific databases, such as the WHO and the European international official sites, were considered in addition. Original articles, including animal and *in vitro* studio, reviews, and epidemiological studies were selected, focusing initially on those published in the last 10 years. Initially, the identified records were imported into Mendeley. After removing duplicates, reviewers separately screened the included papers found through the literature search by title and abstract, using the eligibility criteria. For specific topics when literature was scarce, we included even older publications. The following keywords were used for the search: thyroid hormones, TSH, fT3, fT4, T3, and T4, thyroid, thyroid gland, thyroid dysfunction, thyroid function, endocrine disrupting chemicals, EDCs, BPA, BPS, BPF, phthalates, pesticides, pollutants, air pollution, water pollution, Particulate matter (PM)_2,5_, PM_10,_ heavy metals, climate changes, nutrients, diet, covid19, infections, pregnancy, prenatal, perinatal period, infancy, adolescence, adult life, that were differently matched for all required strings (e.g., **Figure 1**).

### 2.2 Data extraction

Following title and abstract screening, relevant papers were further evaluated. To collect data from the selected papers, a predefined data extraction strategy was used. The extracted data included information such as authors, year of publication, title, abstract, contaminants analyzed, research domain, and study type. Finally, the remaining articles were evaluated after reading the complete content. Furthermore, one author conducted additional screening and data extraction for the most recent publications.

### 2.3 Narrative synthesis

A narrative synthesis was conducted to organize the search findings. To identify the most recent problematic points, the synthesis focused on specific environmental factors and contaminants that influence thyroid development and function.

## 3 Effects of environmental pollutants on thyroid function and disease

Climate change, driven by human activities such as deforestation and burning fossil fuels, has far-reaching consequences for health. Rising temperatures, air pollution, extreme weather events, and changes in disease patterns represent current direct and indirect threats to child health, particularly to the thyroid gland.

The US Environmental Protection Agency (EPA) defines pollution as “any substances in water, soil, or air that degrades the natural quality of the environment; offends [the senses]; causes a health hazard; or [impairs] the usefulness of natural resources” (19). The WHO considers air pollution as a major environmental health issue that affects everyone in low-, middle-, and high-income countries (13). Also, European paediatric scientific societies are highly concerned about the influence of environmental pollution on child health (20). Both the European Society for Paediatric Endocrinology (ESPE) and the European Society for Endocrinology (ESE) are collaborating on a common project, the EndoCompass project (https://www.ese-hormones.org/what-we-do/research/endocompass-european-research-roadmap/), that among many other aspects considers the effect of environmental contaminants, pollution, and climate change.

Air pollutants mainly include particulate matter (PM), ozone pollution (O3), carbon monoxide (CO), nitrogen oxides (NO2 and NOx), and sulfur dioxide (SO2). PM is a mixture of suspended particles with different chemical compositions, classified by size (PM_10_ and PM_2.5_) (12); in particular, PM_2.5_ particles differ from other PM particles having a higher penetration capacity and reactivity, so that PM_2.5_ has become the most relevant air pollutant causing the greatest threat to global public health (13).

Nitrate is a common contaminant of drinking water, particularly in agricultural areas, due to the usage of nitrogen-containing fertilizers. High amounts of nitrate might also be present in some fruits and vegetables (21).

Heavy metals are naturally present in the environment, although their concentrations vary a lot in different geographic areas. They include both essential metals, required micro-nutrients for biological processes (Fe: Iron, Cu: Copper, Zn: Zinc, Se: Selenium, etc.), and toxic chemicals that may damage cell biology and promote malignant transformation (As: Arsenic, Cd: Cadmium, Hg: Mercury, Ni: nickel, etc.). In recent decades, metal pollution of the environment has increased worldwide (22). Water, soil, and atmosphere are vehicles that may be enriched with heavy metals due to their proximity to sources, including natural emissions (non-anthropogenic) and pollution secondary to human activities (anthropogenic). Industry, agriculture, and technology are activities related to higher heavy metal exposure (23).

There are strong links between environmental pollution and adverse health effects in humans; children, in particular, are vulnerable at every level of development (24). Studies have focused mainly on allergic diseases related to increased allergen production, infectious diseases, respiratory diseases, diabetes, and cardiovascular diseases (25).

The following paragraphs focus on the possible associations of environmental pollution on thyroid function in the different ages of life.

### 3.1 Climate and thyroid function

Climate change is a global issue that affects environment, from increased temperatures, floods, droughts, and wildfires to sea level changes and loss of biodiversity. Heatwaves can lead to dehydration, heat exhaustion, endocrine imbalance, and even heatstroke. Rising temperatures can disrupt the thyroid’s ability to maintain hormonal balance. Researchers have observed an association between outdoor temperature and thyroid hormone levels, with increasing TSH and fT3 during autumn-winter periods and decreasing of these during spring-summer periods (26).

Climate change contributes to the release of pollutants and chemicals into the environment, as nitrates. Several studies have shown that the climate significantly impacts the process of nitrate accumulation in plant tissue, with nitrate concentration being lower in years with high rainfall. Seasonal heavy rains wash away soil minerals, while temperature spikes contribute to elevated nitrate and nitrite concentrations; moreover, high temperatures induce moisture loss in fruits and vegetables, all of these amplifying nitrate and nitrite levels in agricultural products, especially vegetables (27), which have a negative impact on the thyroid gland. Also, increased volcanic activity in recent years is associated with a higher risk of thyroid cancer, which will be further described below (23, 28).

Global warming also causes the evaporation of iodide from seawater, and in regions distant from the coast, it results in a depletion of iodide in plant foods and drinking water and iodine deficiency (29). Furthermore, using data-mining techniques, it has been shown that nowadays, climate variables in the aridity index and precipitation, soil leaching, and pH decrease, all predict soil selenium losses that could increase the prevalence of selenium deficiency (30), increasing in turn serum TSH levels.

### 3.2 Effects of particulate matter exposure on thyroid function

Only a few studies have investigated, to date, associations between PM exposure and thyroid function during childhood.

In the newborn, data is not only scarce but also conflicting. The ENVIRONAGE birth cohort study (N:499 newborns) focused on PM_2.5_ exposure during the third trimester of pregnancy, reporting an inverse association between exposure and cord blood TSH levels and FT4/FT3 ratio (31). At variance, other studies reported that prenatal exposure to PM_2.5_ and PM_10_ air pollution was associated with higher newborn total T4 concentrations (32, 33). Howe et al. identified months 3 to 7 for PM_2.5_ exposure and 1 to 8 for PM_10_ exposure of pregnancy as critical windows of exposure, which have been associated with higher thyroxine levels (32). Irizar et al. also suggested that exposure to PM_2.5_ during pregnancy was positively associated with TT4 levels in newborns: TT4 levels collected 3 days after birth increased by 0.206 μg/dl for each 1 μg/m^3^ of increase in exposure to PM_2.5_ during pregnancy. This study analyzed but found no association between NO_2_ exposure levels and TT4 levels at birth (33). The apparent difference between the results of these studies is unclear, and probably due to differences in assay methods as most of T4 is bound to serum transport proteins, such as thyroxin-binding globulin (TBG), and free T4 makes up only for a small fraction (0.03%) of TT4 (34).

The findings in newborns/infants are, however, in agreement with the data collected in adults, as shown in the Di@bet.es study (35), where a significant negative association between exposures to PM_2.5_ and thyroid hormone levels was described, moreover, with relatively low PM_2.5_ concentration ranges, well below the existing European Ambient Air Quality Directive target values (PM_2.5_<25 µg/m^3^) (20). To date, the WHO guidelines recommend a maximum annual PM_2.5_ exposure < 5 µg/m^3^ (16).

There are no published data on the effects of other pollutants on thyroid function in the pediatric age (36).

### 3.3 Effects of exposure to nitrates and nitrites on thyroid function

There are limited studies that have investigated NO_2_ pollution and thyroid hormone levels at birth, without observing any significant association (32, 33). However, one study from China found that higher maternal NO_2_ exposure was linked to the risk of congenital hypothyroidism in newborns (37), but further research is needed.

According to WHO, drinking water is the most likely primary source of nitrate exposure. Another source can be vegetables and fruits when nitrate concentrations are > 10 mg/L (38). Studies from Bulgaria, Slovakia, Germany and the USA have reported associations between nitrate exposure and thyroid function in different age periods: some of these have reported that school children and pregnant women exposed to high nitrate levels in drinking water (75 mg/L) had a higher risk of developing goiter and thyroid disorders than school children and pregnant women exposed to low nitrate levels (8 mg/L) (39–42). Another study conducted in Slovakia school children described a significantly greater thyroid volume in children from high-exposure nitrate areas (> 200 mg/L) compared with children of comparable age from low-exposure nitrate areas (< 2 mg/L). No differences in the levels of T3 or T4 were found, but a higher prevalence of TSH levels in the range > 4.0 mIU/L was observed, in addition to increased anti-thyroid peroxidase antibodies in the children living in high-exposure nitrate areas. These studies confirmed excessive nitrate levels in drinking water might be a risk factor for thyroid dysfunction in vulnerable population groups (40, 41).

### 3.4 Relationships between environmental pollution and thyroid cancer

The global incidence of thyroid cancer has increased rapidly, with a rate of +1.1% per year in the last decades (43–45). In fact, according to the Surveillance, Epidemiology, and End Results (SEER) program, new cases of thyroid cancer in people under the age of 20 years account for 1.8% of all thyroid malignancies diagnosed. Among the pediatric population, adolescents seem to have a 10-fold greater incidence than younger children, with a female-to-male proportion (5:1). In particular, in 15- to 19-year-old adolescents, thyroid cancer represents the eighth most frequently diagnosed cancer, and the second most common cancer among girls (45). However, a large geographical heterogeneity in incidence rates has been evidenced. Epidemiology and country-specific trends in children and adolescents seem similar to those highlighted in adults. Although some authors suggest that this might account for overdiagnosis in the pediatric population (43), the hypothesis of environmental factors being of importance for thyroid cancer must be taken into serious consideration. Currently, it is well known that exposure to some environmental pollutants, such as radioiodine, plays a role in carcinogenesis. Following the Chornobyl disaster, in fact, areas next to the nuclear power plant registered an increase in thyroid cancers; the Belarus pediatric population, in particular, was screened and presented a rapid increase in thyroid cancer incidence in children after 1986, especially in the most radionuclide contaminated areas (44, 45).

The International Agency for Research on Cancer (IARC) has classified outdoor air pollution and particulate matter (PM) in outdoor air pollution as carcinogenic to humans, and in accordance with the WHO, in 2013, particulate matter air pollution was declared as carcinogenic (30). Air pollution contains several chemicals capable of producing genomic instability, which is an underlying feature of cancer development (46, 47). The mechanism by which PM_2.5_ might cause thyroid cancer is unclear; probably, it is related to volatile organic compounds that are bound to particulate matter that might penetrate the bloodstream through the alveolar barrier.

Both a study conducted in the US (48) and one conducted in Brazil (49) showed an association between particulate matter and the development of thyroid cancer; a 10 μg/m^3^ increase in PM_2.5_ concentration over 12, 24, and 36 months was associated with a greater likelihood of being diagnosed with Papillary thyroid carcinoma (PTC), with this likelihood increasing with increasing duration of exposure to PM_2.5_. Additionally, a study of 550,000 patients in China found that emissions from industrial waste gas (consisting mainly of particulate matter, sulfur dioxide, and nitrogen dioxide) were associated with increased thyroid cancer (50). At variance with these findings, Park et al. found that exposure to particulate matter was negatively associated with the incidence of thyroid cancer (51). These conflicting findings could be due to the fact that this study did not discuss the methods used to measure the levels of particulate matter, and it was not clear how these levels were linked to each patient, limiting the generalizability of these findings.

A further study demonstrated that the probability of developing papillary thyroid cancer increased with a longer duration of PM_2.5_ exposure: a 5 μg/m^3^ increase of PM_2.5_ concentrations over 12 months of exposure was not associated with the incidence of PTC, but the odds of developing PTC after a 5-μg/m3 increase in PM_2.5_ exposure was increased by 18% in 24-month and by 23% after 36-months of exposure (52).

Heavy metals are considered among known carcinogenic environmental pollutants. The carcinogenic effect of metals on target cells can occur through several mechanisms, such as causing changes in bioavailability and intracellular distribution of peptides by interacting with intracellular proteins, enzymes, and other cell components. The possible carcinogenic mechanisms involve an increase in oxidative stress, that may cause oxidative DNA damage, interfere with DNA repair systems, deregulate growth control mechanisms, and modify DNA methylation patterns (30).

Living in volcanic areas represents a further risk factor as some of the highest incidences of thyroid cancer are registered in volcanic regions (53, 54), and these areas have higher concentrations of heavy metals. However, the causative role of heavy metals is far from being properly understood. To date, in fact, the dose and duration of exposure to heavy metals that might be harmful are not well defined. For some heavy metals (Cd, Hg), for which carcinogenic role is known, there are no specific exposure levels that indicate toxicity, as toxicity is not related only to environmental concentrations, but depends on other mechanisms also that have not yet been taken into consideration (30, 55). Furthermore, exposure to a combination of heavy metals rather than a single could account for the toxicity (56, 57). This concept is generally valid for environmental exposure to pollutants as each individual is exposed to multiple pollutants that react with the human body chemical environment, and the final effect is currently largely unknown.

Many heavy metals (As, Cd, Cr, Hg, and Pb) have never been tested yet as potential human thyroid carcinogens. Studies have highlighted negative associations between thyroid volume and Cr, Se, and Zn contents in the hair samples of children. In the meantime, a link between thyroid volume and Pb and Mn concentrations has been proposed, presuming that these metals may interfere with the thyroid gland either directly or indirectly by influencing iodine intake or thyroid hormone and TSH levels (58, 59).

Volcanic activity is also related to an increase in environmental Radon concentrations, although a specific association of thyroid cancer with Radon levels has never been put forward (60). Belonging to the same ethnic group was also demystified as a cause of the increased incidence. In fact, people from volcanic islands residing in non-volcanic areas were unaffected (61).

Thus, studies to date regarding the role of environmental pollution with respect to thyroid function are still scarce, especially in childhood. Therefore, further studies are needed, investigating both the correlation in different age groups and studies to identify the pathogenetic mechanisms by which pollutants affect the thyroid gland.

## 4 The effects of endocrine-disrupting chemicals on thyroid function

EDCs can enter the environment through manufacturing processes, agricultural practices, waste disposal, etc. Contamination can occur through skin contact, food digestion, inhalation of contaminated products, or by vertical transmission via the placenta. EDCs exert their effects through multiple mechanisms. Some chemicals can bind to hormone receptors in the body, either activating or inhibiting their function. Others can interfere with hormone production, transport, metabolism, or clearance. In addition, they may have other modes of action, as causing oxidative stress, genetic susceptibility, and epigenetic modifications (e.g., DNA methylation, miRNA) (17, 62, 63), ultimately resulting in gene expression changes (62, 64, 65) in particular when exposure occurs during prenatal and early postnatal life.

Specifically, some studies have demonstrated that EDCs such as bisphenol A (BPA), di-2-ethylhexyl phthalate (DEHP), dibutyl phthalate (DBP), and pesticides exert some of their endocrine-disrupting activity by influencing methylation patterns. Furthermore, polychlorinated biphenyls (PCBs), BPA, and phthalates have been found to induce or inhibit the expression of other epigenetic regulating systems, as the miRNA network. Tributyltin and polycyclic aromatic hydrocarbons (PAHs) bind to Peroxisome proliferator-activated receptors (PPARs), modifying both its methylation status and the expression of its target genes, ultimately affecting insulin sensitivity. Furthermore, exposure to all of these EDCs can impact the expression and enzymatic activity of DNA methyltransferases, potentially interfering with enzymes like aromatase (65), ultimately modifying estrogen and androgen content. Importantly, changes to the epigenome induced by exposure to EDCs may have transgenerational effects, influencing health and disease across multiple generations even after the initial exposure has occurred (63–65).

The potential impact of EDCs on thyroid hormone regulation in children is of particular concern as thyroid hormones play a crucial role in the growth and central nervous system development, and control the overall physiological homeostasis (64). The fetal thyroid gland is underdeveloped during the first half of pregnancy, relying exclusively on maternal thyroid hormones, particularly T4, which crosses the placenta, thus, normal brain development requires a perfect thyroid hormone balance. Moreover, congenital hypothyroidism, or changes in thyroid hormone levels during pregnancy and at birth, can cause permanent neurodevelopmental disorders in infants (64, 66, 67).

**Figure 2:**
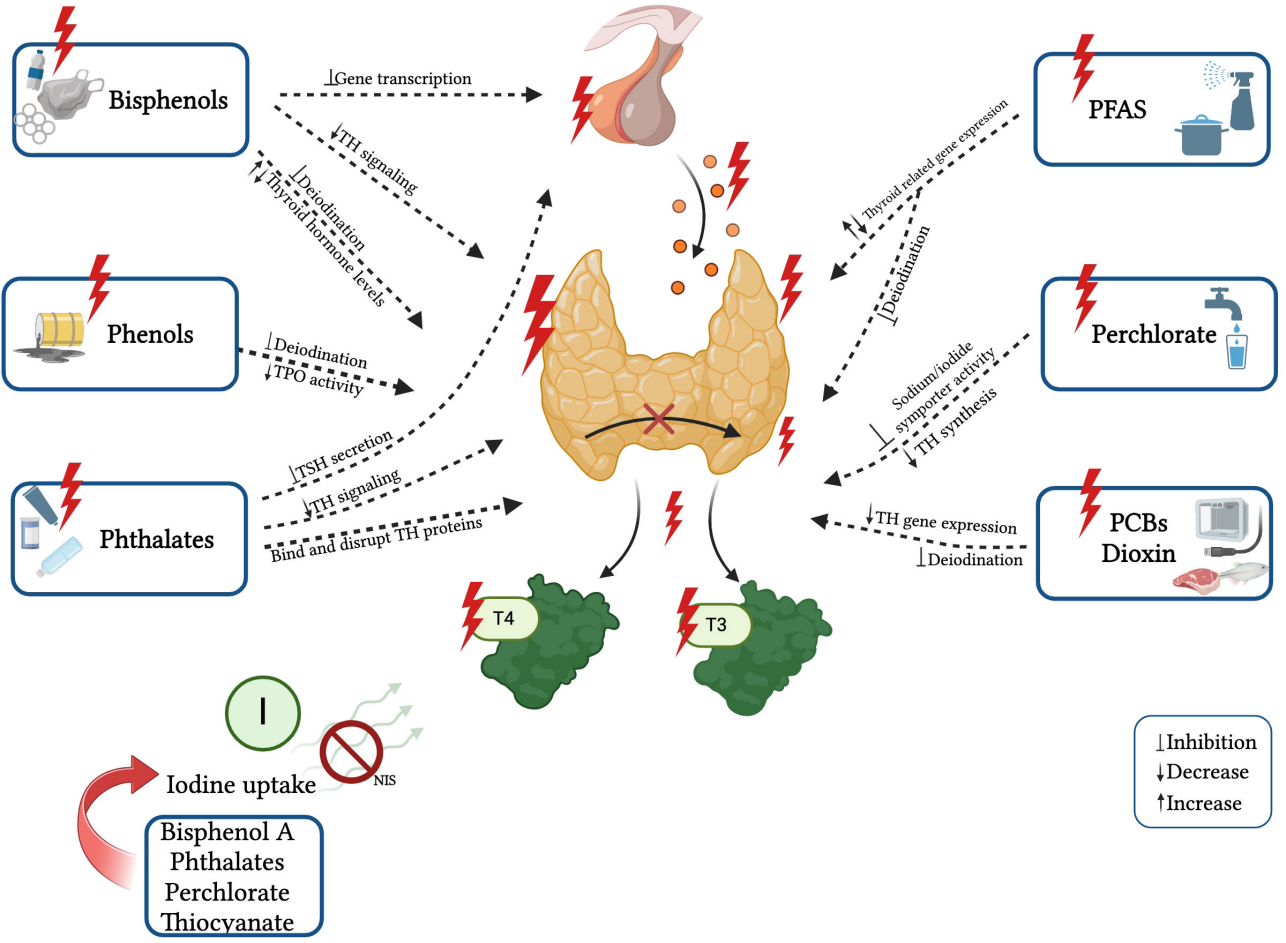
Known effects and mechanisms of action of single EDCs on thyroid function. Created with BioRender.com.

In recent years, there has been increasing evidence of the effects of EDCs exposure on thyroid hormone levels from animal experiments and epidemiological studies. Structural similarities have been shown between some EDCs and thyroid hormones (THs) (64, 68, 69), and it has been proven that they can disrupt the THs synthesis, release, action on target tissues, and transportation by displacing the hormones from their binding proteins or by changing the content/activity of transporters. These EDCs include bisphenol A (BPA), phthalates, perchlorate, dioxin, polychlorinated biphenyls (PCBs), polybrominated diphenyl ethers (PBDEs), organophosphate pesticides (OPs), per- and polyfluoroalkyl substances (PFAS), and triclosan (20, 68). They can dysregulate the HPT axis, potentially leading to the development of thyroid gland hypertrophy and hyperplasia, hypothyroidism or hyperthyroidism, and even thyroid cancer (20, 64, 69, 70). The known effects of single EDCs on thyroid function are reported in **Figure 2**.

### 4.1 The effects of bisphenols and phthalate metabolites on thyroid function

Bisphenol A and phthalates that are prevalent in plastics and personal care products have been correlated with fluctuations in thyroid hormone levels, disturbances in thyroid-stimulating hormone (TSH) regulation, and with hindered development of the thyroid gland (20, 64, 66, 71). BPA, phthalates, perchlorate, and thiocyanate decrease thyroidal iodine uptake by competitively inhibiting the sodium-iodide symporter (NIS) (64, 72, 73). Moreover, bisphenols can modify thyroid hormone action by antagonizing thyroid receptor action, altering gene expression, and mimicking thyroid transport proteins (17, 74).

Chao Xiong et al. reported that maternal BPA exposure during the first trimester of pregnancy was associated with higher neonatal TSH levels and that BPS exposure throughout pregnancy was also associated with TSH levels in newborns. In addition, co-exposure to both bisphenols during the first trimester of pregnancy was significantly associated with neonatal TSH levels, and females might be more susceptible (75). A further prospective cohort study defined the impact of maternal exposure to bisphenols (BPs) on offspring’s thyroid hormone levels in cord blood; BPA exposure was associated with lower total thyroxine (TT4) and free triiodothyronine (fT3) concentrations, BPS exposure with lower TSH in boys, and BPAF exposure with higher TT3 and fT3, particularly in girls. The exposure to a mixture of BPs was associated with higher TT3 levels and fT3 in both sexes. The mechanisms involved and affected by these bisphenols are represented by transcriptional changes in thyroid hormone synthesis genes and changes in related gene transcription, and impaired iodine uptake, affecting overall thyroid function (76). The most affected genes were thyroid hormone synthesis genes such as SLC5A5 and Thyroid Peroxidase (TPO) genes, and transcription genes PAX8, FOXE1, and NKX2–1 (77).

Thyroid volume was described to be increased with age after BPA exposure in 718 Chinese, 9–11 year-old children, and the incidence of multiple thyroid nodules also increased with age (78). The longitudinal SMBCS study found that higher maternal urinary BPA levels during pregnancy were associated with increased fT4 levels in cord serum and predicted a high risk of behavioral difficulties in children at 10 years of age, particularly in boys (79).

When considering phthalates, exposure to di-(2-ethylhexyl) phthalate (DEHP) metabolites in rats was mainly associated with altered pituitary TSH secretion and disrupted thyroid hormone homeostasis with changes in receptor expression levels (80). Furthermore, *in vivo* studies have shown that the complex effects of DEHP and BPA mixtures may lead to reduced thyroid weight, which ultimately would affect the development of the thyroid gland (81). In humans, instead, some studies suggest that individual maternal urinary phthalate metabolite concentrations would have no effect on cord serum TSH levels (72, 82), but the data are controversial. Huang et al. reported that maternal phthalate levels in the second trimester of pregnancy, were positively associated with T3 and fT4 levels in cord serum (83). Moreover, phthalates antagonize the binding of T3 to thyroid receptor-β, limiting T3 cell uptake, and disrupting sodium-iodine transporter transcription, lowering thyroxine (T4) and T3 levels in pregnant women and children (84, 85).

The Columbia Center study showed that maternal urinary mono-(2-ethylhexyl) phthalate (MEHP) levels were positively linked with fT4 in children at age 3 years (24). Moreover, there is a link between early childhood phthalate exposure and thyroid function from 6 to 12 years of age. Two further recent studies have shown, that phthalate exposure was associated with decreased TSH and increased T3 in children (85, 86). Researchers have also described an overall positive association of phthalate mixtures with TT3 serum levels in adolescents (85, 87, 88).

A large US study in 356 adolescents aged 12–19 years showed a positive correlation between TT3 and TSH with mono‐(carboxyisoctyl) phthalate (MCOP) and a negative correlation of TSH with MCNP. Moreover, elevated levels of phthalates in pubertal boys and girls were associated with higher DNA methylation levels at the thyroid hormone receptor interactor 6 (TRIP6) promoter region, a gene associated with pubertal onset (87, 88). A further recent longitudinal study of 166 children described a positive relationship between mono (2-ethyl-5-hydroxyhexyl) phthalate (MEHHP) and fT4, and a negative relationship between mono-methyl phthalate (MMP) and T3, T4, and fT4 serum levels (89). Besides, DEHP metabolites have been reported to be positively associated with TT3, and MCPP to be negatively associated with fT4 and TT4 in teenagers aged 12 to 19 years of age (88).

### 4.2 Effects of perchlorate, dioxin and persistent organic pollutants on thyroid function

Perchlorate, commonly present in both drinking water and specific food items, competitively hinders the absorption of iodine by the thyroid gland, subsequently reducing the synthesis of thyroid hormones, and has been related with decreased cognitive function in offspring (89).

Prenatal exposure to dioxin (TCDD exposure) has been reported to be associated with changes in thyroid function in 2-year-old children born to women living in Seveso where an explosion that occurred 40 years ago has yet health implications, and lower fT3 and fT4 levels are observed in the children born to these mothers compared with the general Italian population (90). In 2 to 5-year-old children, prenatal dioxin exposure can increase TSH and fT4 levels, which were found especially in males (91).

Persistent organic pollutants such as PBDEs and PCBs showed associations with reduced serum thyroid hormone levels, altered expression of TH-responsive genes, modifications in thyroid hormone-binding proteins, and an elevated likelihood of hypothyroidism (20, 64, 66, 71).

In the neonatal period, prenatal exposure to dioxin-like contaminants has been described to be associated with higher neonatal free thyroxine (fT4), especially in boys. Researchers didn’t find any significant relationship between total PCBs or non-dioxin-like PCBs exposure and any maternal or neonatal thyroid hormone, implying that non-accidental PCB exposure caused thyroid damage primarily through dioxin-like action (89).

### 4.3 Effects of exposure to organophosphate pesticides, per- and poly-fluoroalkyl substances, on thyroid function

Organophosphate pesticides decrease thyroid hormone levels and modify thyroid hormone metabolism. Experimental *in vivo* studies have shown that the changes in TH levels cause decreased circulating transthyretin, affecting the hepatic metabolism of T4, inducing histological changes in the thyroid gland, and reducing brain weight in the offspring (20, 66, 71, 92).

Per- and poly-fluoroalkyl substances (PFAS) have been shown to reduce circulating thyroid hormone levels by interfering with intracellular signaling cascades, like mitogen-activated protein kinase (MAPK) pathway, which can alter thyroid hormone production, secretion, and cellular responses (93). Thyroid hormone levels can be modified by PFAS exposure both prenatally and postnatally, and higher PFAS mixture concentrations in babies have been found to be associated with lower total thyroxine levels (74, 94), but not with TSH levels in neonates (95). The flame retardant DE-71 (a mixture of polybrominated diphenyl ethers) has been reported to inhibit human differentiated thyroid cell function *in vitro*, to inhibit Thyroid globulin (Tg)-release from TSH-stimulated thyrocytes, and to inhibit the expression of mRNA encoding for Tg, TPO, and TSHr (96). A comprehensive meta-analysis from thirty-two cohort studies has investigated the relations between prenatal exposure to organochlorine, PFAS, and other EDCs, levels of maternal thyroid hormones during pregnancy, and neonatal thyroid hormone levels describing negative associations of organochlorine and PFAS exposure and neonatal TT4 levels, suggesting potential adverse effects (66, 97); the authors hypothesized a change in the balance between thyroid hormone biosynthesis and elimination (97) an induction of microsomal enzymes as uridine diphosphate glucuronosyltransferase, and an increased T4 to T3 conversion by inducing deiodinase (98, 99).

### 4.4 Effect of exposure to other EDCs on thyroid function

Thyroid peroxidase (TPO) activity can be inhibited by thiocyanates and phenols as they affect the conversion of iodide to iodine and subsequently coupling processes inhibiting the production of thyroid hormones and causing altered thyroid homeostasis (70, 73). Phenols, especially in baby girls, are associated with TSH levels, but not with total TT3 and fT4 during the pregnancy and at birth. This leads to the hypothesis that genetic variants in the Iodothyronine Deiodinase 1 (DIO1) and Iodothyronine Deiodinase 2 (DIO2) genes can modulate these associations (100). Prenatal triclosan exposure was found to affect Thyroid Peroxidase Antibodies (TPOAb) levels in three-year-old children, resulting in 328% TPOAb higher levels (101). Notably, populations with high thyroid antibody status appear to exhibit increased susceptibility to the effects of *in-utero* exposure to thyroid-disrupting chemicals, emphasizing the potential long-term impact of such exposures on thyroid health (90).

Interestingly, phytoestrogens, which also act as EDCs, have an influence on thyroid hormones in adolescents as well. Yun Fan et al. reported that elevated levels of the urinary phytoestrogen enterolactone (ENT) were positively associated with TSH levels in 12–19-year-old girls and with TSH and TT3 in this age category in boys. Moreover, equol (EQU) levels were negatively associated with TT4 in both sexes. So, we need to monitor phytoestrogen intake via meals as well (102).

Finally, some effects are sex-specific, as previously described for bisphenols, phthalates, and PCBs in boys (76, 79, 89), and as for triclosan, parabens, and OH-MPHP, negatively associated with T4, in girls (103).

It should also be mentioned that in the near future machine learning will be of great help to identify chemicals that interfere with the endocrine system. A recent study has highlighted using these techniques a few new chemicals predicted to interfere with the TSH receptor (104).

The currently known effects of single EDCs on thyroid function are reported in **Table 1**.

**Table 1:** The known effects of single EDCs on thyroid function.

EDC	Window of exposure (age)	Findings and effects	References
Bisphenols	Prenatal exposure and neonatal period	↑ TSH in neonates	(75)
	Prenatal exposure and in newborns	BPA - ↓TT4 and fT3 BPS - ↓ TSH in boys BPAF - ↑ TT3 and fT3 in girls BPs - ↑ TT3 and fT4	(76)
	9–11 year old children	BPA - ↑ thyroid volume ↑ incidence of thyroid nodules	(78)
	Prenatal exposure and 10 year old children	BPA - ↑ fT4 in cord serum ↑ risk of behavioral difficulties at school age	(79)
Phthalates	Prenatal exposure and newborns	No effect on cord serum THs levels, but for each 10-fold ↑ in MEP one observes ↓TT4 in neonates, and for a 10-fold increase in MBzP one observes ↓ TSH in 19% of neonates	(72)
	Prenatal exposure and 3 year old children	No association with TSH levels, but maternal MEHP vevels were positively associated with fT4 in children at age 3 yr	(82)
	Prenatal exposure and newborns	MEP and MiBP - ↑ T4, and ↑ fT4 ↓ T3 and MiBP and MEOHP — ↓ TSH	(83)
	Adolescents 12–19 year olds	MCOP - ↑TT3 and MCNP - ↓ TSH	(85)
	Prenatal exposure and 6 year olds	MnBP - ↓ TSH and ↓ fT4 × TSH	(86)
	Adolescents 12–19 year olds	Positive association of phthalates with T3 serum levels, MCOP with TT3 and TSH, and negative correlation of TSH with MCNP. DEHP positively associated with TT3, and MCPP was negatively associated with fT4 and TT4	(88)
Dioxin-like contaminants and PCBs	Prenatal exposure and neonatal period	↑ fT4 in neonates	(89)
Dioxin	Prenatal exposure and 2 year olds	↓ fT3 and fT4	(90)
	Prenatal exposure and up to 6 year olds	↑ TSH and fT4, especially in boys	(91)
PFAS	Prenatal exposure and newborns	↓ fT4	(95)
Phenols	Prenatal exposure and neonatal period	↓ TSH levels	(100)
Triclosan	Prenatal exposure and up to 3 year of age	↓ TT4 cord levels, but disappear later ↑ TPOAb levels in 3yr old children	(101)

↓, decrease; ↑, increase.

## 5 Effects of diet, nutrition, food pollution and microplastics on thyroid function

The relationship between diet and thyroid health plays a crucial role in maintaining hormonal balance and preventing thyroid dysfunctions. A nutrient-rich dietary style, including essential elements such as iodine, selenium, iron and vitamin D, can promote the proper synthesis of thyroid hormones and regulate their activity. Moreover, there are currently no dedicated nutritional recommendations for patients with thyroid diseases. Existing guidance relies solely on the opinions and advice of individual specialists, often providing incomplete and sometimes contradictory information. Recent studies have demonstrated that high consumption of animal fats can induce an increase in the production of thyroid autoantibodies (105). The saturated fatty acids found in animal fats can indeed trigger a pro-inflammatory response through the activation of cytokine transcription. Studies in rats have confirmed that excessive consumption of animal fats determines thyroid dysfunction and may contribute to the pathogenesis of hypothyroidism (106). A study conducted by Ruggeri et al. on euthyroid patients with Hashimoto’s disease showed that markers of oxidative stress (evaluated through advanced glycation end-products - AGEs) were significantly higher in patients compared to controls, and the activity of the enzymes glutathione peroxidase and thioredoxin reductase, as well as plasma antioxidant activity, was reduced in the patients compared to controls. When evaluating the dietary habits of the enrolled cohort, it emerged that patients consumed animal-derived foods (meat, fish, and dairy) more frequently compared to the control group, which preferred a diet richer in plant-based foods (legumes, fruits, vegetables) (107). This suggests the positive influence of a low intake of animal foods on the oxidative-antioxidant balance. An anti-inflammatory diet, rich in vitamins, minerals, polyphenols, and phytosterols, seemed to be able to reduce the circulating levels of thyroid autoantibodies (108). Natural antioxidants as vitamins A, C and E are found mostly in plant-based products, including a wide variety of fruits and vegetables. These studies highlighted the importance of recommending a Mediterranean diet as an adjuvant in the treatment and prevention of thyroid autoimmunity, thanks to its antioxidant properties (107, 108).

Diet can also influence the composition of the microbiota. Several studies have illustrated the change in microbiota composition following dietary modifications. The intestinal bacterial flora plays a crucial role in maintaining metabolic, nutritional, and even immunological homeostasis (109). The hypothesis of a thyroid-gut axis is becoming increasingly concrete. In addition to influencing the absorption of minerals important for thyroid function (iodine, selenium, zinc, and iron), the microbiome is involved in endogenous and exogenous thyroid hormone metabolism. The presence of iodothyronine deiodinase, an enzyme that converts T4 into its active T3 form or into reverse T3 (rT3), has been found in the intestinal wall, thus influencing total body T3 levels (110). A review by our group reported how the microbiota plays a role in metabolizing endocrine disruptors introduced through diet, by means of a bidirectional interaction that leads to dysbiosis and changes in pathways involved in the development of various metabolic diseases (63). Dysbiosis and the subsequent negative influence on the immune system and intestinal permeability can promote the development of inflammatory and autoimmune diseases, including thyroid diseases (110–112).

Food pollution may modify these factors, which will, in turn, affect thyroid function.

As we described, food may be exposed to endocrine-disrupting chemicals through pesticides, fertilizers, canned products, and plastic particles that exhaust and alter thyroid function (17). Ultra-processed food is an industrially manufactured product taken from natural foods or synthesized from other organic components (113). Its consumption could also change thyroid function. A unique prospective cohort study by Zhang et al. found that ultra-processed food consumption was associated with higher risk of subclinical thyroid dysfunction in adults (114).

Another concern is related to the increasing global prevalence of nitrate and nitrite accumulation in vegetables and fruits owe to climate changes (29). As nitrates and nitrites in the human body undergo metabolic conversion into nitric oxide, higher levels can disrupt thyroid function. Nitrate can competitively interfere with iodine uptake by the thyroid gland and influence hormonal synthesis. Moreover, meat consumption with higher levels of nitrates/nitrites can stimulate the synthesis of endogenous N-nitroso compounds. Both nitrosamines and N-nitroso compounds have carcinogenic effects (42, 115). A meta-analysis concluded that a dietary high nitrate intake was connected with an increased risk of thyroid cancer (odds ratio [OR] = 1.40, 95% confidence intervals [CI]: 1.02, 1.77) (116).

Finally, the accumulation of nano- and microplastics in meat and fish can lead to chronic exposure and accumulation of these in tissues. It is hypothesized that microplastics may disrupt the epithelial barrier and alter immunological responses (63). Animal studies have described that chronic exposure to polystyrene nanoplastics inhibits serum T3 and circulating levels of thyroid hormones, which significantly elevates TSH (117).

Additionally, contaminated seafood and seafood products can contain heavy metals like mercury, which are bioaccumulated in the thyroid cells and can contribute to the development of autoimmune thyroiditis, hypothyroidism, and thyroid cancer (118).

## 6 The effect of infectious agents on thyroid function: lessons from the COVID-19 pandemic

The risk of infections and their effects represents one of the biggest environmental threats and planetary emergencies to date, as shown by the COVID-19 pandemic.

Since December 2019, the Coronavirus Disease 2019 (COVID-19), caused by the etiologic agent Severe Acute Respiratory Syndrome Coronavirus-2 (SARS-CoV-2), has been the cause of high mortality and morbidity all over the world. At the end of May 2023, over 767 million human infections and over 6.9 million deaths have been reported worldwide (119).

The pediatric population, instead, since the beginning of the pandemic, has been less affected than adults, with a global milder clinical presentation and low rates of hospitalization and death. However, rare and severe complications may also develop in children, with a systemic involvement due to a multisystem inflammatory syndrome (MIS-C) (120).

SARS-CoV-2 is an enveloped RNA virus that belongs to the Coronaviridae family (Nidovirales order). Its genome is a single-stranded positive-sense RNA that contains four structural proteins: spike protein (S), membrane protein (M), nucleocapsid protein (N) and envelope protein (E) (121). The spike protein is a viral surface protein and consists of two subunits (S1 and S2). It is involved in the viral attachment and in the host cell entry: the S1 subunit is able to bind the angiotensin-converting enzyme 2 (ACE2) receptor while, in a second step, the S2 subunit allows the virus to enter the cell by membrane fusion (122).

ACE2 is widespread in different organs, including the respiratory and gastrointestinal tract, the brain, the kidneys, the heart, and vessels, and this is the reason why, although the main viral target still remains the respiratory epithelium, SARS-CoV-2 can potentially infect multiple tissues (123). Moreover, ACE2 is also expressed in several endocrine tissues, such as the ovaries, the testis but also the hypothalamus, the pituitary, and the thyroid gland, with a consequent possible impact of the infection on endocrine function (124). SARS-CoV-2 can attack, in fact, thyroid follicular cells via ACE2 with possible consequences on thyroid function (125). In addition, besides the direct invasion of the thyroid tissue, indirect mechanisms can also alter thyroid function. First, both the hypothalamus and the pituitary gland, which regulate the thyroid axis, express ACE2 receptors, being additional targets of the viral infection. Second, it is worth noticing that thyroid hormones are strongly linked to the regulation of the immune system (126). Patients with severe forms of COVID-19 infection characterized by a hyperinflammatory state due to the cytokine storm may develop an acute hypothalamus/pituitary/thyroid axis dysfunction that may cause both acute hypothyroidism and immune-mediated damage (127).

In critical patients, euthyroid sick syndrome or non-thyroidal illness syndrome, characterized by decreased fT3 but normal TSH levels, has been described as probably due to the abnormal hyperinflammatory state: in fact, in most cases, this condition was transient and resolved over time (128, 129).

Moreover, a certain increase in incidence of hypo- and hyperthyroidism as *de-novo* diagnosis or exacerbations of pre-existing disorders were described, thus, the indication of routine evaluation of thyroid function in infected patients, in particular in those presenting severe forms of COVID-19 disease was recommended (130, 131).

Considering specifically the paediatric population, it is important to remark that there is still a considerable lack of information on the impact of COVID-19 disease on thyroid function, and all the evidence described above derives from studies conducted on adult patients. In fact, since the beginning of the pandemic, only about 17.9% of the total registered cases concerned children, partially explaining this current lack of data; moreover, children rarely develop severe disease with the hyperinflammatory response, thus protecting them from thyroid acute dysfunctions (132).

Later in the course of the pandemic, after the beginning of the vaccination program, an increasing number of cases of possible alterations in thyroid function after the administration of COVID-19 vaccines were reported, in parallel with reassuring data on global safety and efficacy.

The most common suspected adverse event reported was subacute thyroiditis, followed by Graves disease with other incidental reports of painful thyroiditis, silent thyroiditis, concurrent Graves disease and subacute thyroiditis, thyroid eye disease, overt hypothyroidism, atypical subacute thyroiditis, and painless thyroiditis with thyrotoxic periodic paralysis (133).

Recently, new data on the possible correlation between COVID-19 vaccines and thyroid inflammatory disease have been reported with the confirmation that subacute thyroiditis may be considered an uncommon complication that may follow COVID-19 vaccination. Interestingly, as described by Şendur et al., this adverse effect tends to affect young and middle-aged adult women with a certain genetic background. Conversely, to date, no cases involving children or adolescents have yet been reported (134).

It is worth noticing that the types of COVID-19 vaccinations that most frequently cause adverse effects on thyroid function are mRNA-based vaccines, followed by adenovirus-vectored SARS-CoV-2 vaccines and inactivated vaccines (134). Considering that most of the reported post-vaccine thyroid complications had a favorable outcome with resolution after treatment, the benefits of COVID-19 vaccinations clearly still prevail over the possible development of any thyroid disease.

## 7 Conclusions and future directions

Many environmental factors can influence the development of the thyroid gland and its function throughout life, and there is a need for monitoring and further understanding.

Air and water pollutants, climate change, endocrine-disrupting chemicals, nutrients and food pollutants, and infectious agents can all have a significant effect on thyroid cancer, but current data are still scarce. In particular, we lack information on determinants during childhood and have limited knowledge and understanding of many pollutant mechanisms of action.

The effects of Endocrine Disruptors and thyroid function are currently the most studied among the factors described above, and both epidemiological data and studies of mechanisms of action are available but are yet insufficient and have not covered all ages and critical windows of exposure.

There is a lack of studies, in particular, in the prenatal and perinatal periods, notwithstanding the global attention on the first 1,000 days of life, as *in-utero* exposure to contaminants is known to determine epigenetic changes with transgenerational effects, and to be associated with an increase in non-communicable diseases in later life.

The collective evidence from various studies underscores the intricate relationship between endocrine-disrupting chemicals and thyroid function in children. The complex interplay between EDCs and thyroid function involves many mechanisms. Even minor changes in thyroid hormone functions, especially during critical developmental periods, may have lasting health effects, particularly on neurocognitive development. Therefore, understanding the association between EDCs and thyroid disease in childhood is crucial for developing preventive strategies, promoting regulatory measures, and minimizing exposure to these chemicals, ultimately safeguarding thyroid health.

Infectious diseases currently represent a planetary emergency among others, and many can affect thyroid function; in particular, it has been demonstrated that the SARS-CoV-2 infection is a possible cause of thyroid inflammation and can trigger autoimmune processes in adults. COVID-19 vaccination has been supposed to be able to induce autoimmune thyroiditis in a few young and middle-aged adult women, although the overall advantages of the vaccination still override these possible risks. Data in children and newborns are still lacking.

Future studies should focus on the role of all environmental factors on thyroid function starting from pregnancy, as these will have effects throughout life.

## Author contributions

MS: Conceptualization, Writing – review & editing, Data curation, Formal Analysis, Methodology, Supervision, Writing – original draft. AS: Conceptualization, Investigation, Visualization, Writing – original draft, Writing – review & editing. MP: Data curation, Writing – review & editing. VP: Formal Analysis, Writing – original draft, Writing – review & editing. VD: Investigation, Writing – original draft. AG: Writing – original draft. MG: Investigation, Writing – original draft. MM: Writing – original draft, Investigation. AM: Investigation, Writing – original draft. RR: Investigation, Writing – original draft, Writing – review & editing. SB: Writing – review & editing. LI: Formal Analysis, Writing – review & editing. SE: Writing – review & editing. BP: Supervision, Writing – review & editing.
